# Anthrax lethal toxin and tumor necrosis factor-α synergize on intestinal epithelia to induce mouse death

**DOI:** 10.1093/procel/pwad050

**Published:** 2023-10-19

**Authors:** Xinhe Gao, Teng Teng, Yifei Liu, Tingting Ai, Rui Zhao, Yilong Fu, Peipei Zhang, Jiahuai Han, Yingying Zhang

**Affiliations:** State Key Laboratory of Cellular Stress Biology, School of Life Sciences, Faculty of Medicine and Life Sciences, Xiamen University, Xiamen 361102, China; State Key Laboratory of Cellular Stress Biology, School of Life Sciences, Faculty of Medicine and Life Sciences, Xiamen University, Xiamen 361102, China; State Key Laboratory of Cellular Stress Biology, School of Life Sciences, Faculty of Medicine and Life Sciences, Xiamen University, Xiamen 361102, China; State Key Laboratory of Cellular Stress Biology, School of Life Sciences, Faculty of Medicine and Life Sciences, Xiamen University, Xiamen 361102, China; State Key Laboratory of Cellular Stress Biology, School of Life Sciences, Faculty of Medicine and Life Sciences, Xiamen University, Xiamen 361102, China; State Key Laboratory of Cellular Stress Biology, School of Life Sciences, Faculty of Medicine and Life Sciences, Xiamen University, Xiamen 361102, China; State Key Laboratory of Cellular Stress Biology, School of Life Sciences, Faculty of Medicine and Life Sciences, Xiamen University, Xiamen 361102, China; State Key Laboratory of Cellular Stress Biology, School of Life Sciences, Faculty of Medicine and Life Sciences, Xiamen University, Xiamen 361102, China; Research Unit of Cellular Stress of CAMS, Xiang’an Hospital of Xiamen University, Cancer Research Center of Xiamen University, School of Medicine, Faculty of Medicine and Life Sciences, Xiamen University, Xiamen 361102, China; Laboratory Animal Center, Faculty of Medicine and Life Sciences, Xiamen University, Xiamen 361102, China; State Key Laboratory of Cellular Stress Biology, School of Life Sciences, Faculty of Medicine and Life Sciences, Xiamen University, Xiamen 361102, China

**Keywords:** lethal toxin, TNF, p38α, intestinal epithelial cell, cell death

## Abstract

*Bacillus anthracis* lethal toxin (LT) is a determinant of lethal anthrax. Its function in myeloid cells is required for bacterial dissemination, and LT itself can directly trigger dysfunction of the cardiovascular system. The interplay between LT and the host responses is important in the pathogenesis, but our knowledge on this interplay remains limited. Tumor necrosis factor-α (TNF-α) is a pleiotropic pro-inflammatory cytokine induced by bacterial infections. Since LT accumulates and cytokines, predominantly TNF, amass during *B*. *anthracis* infection, co-treatment of TNF + LT in mice was used to mimic *in vivo* conditions for LT to function in inflamed hosts. Bone marrow transplantation and genetically engineered mice showed unexpectedly that the death of intestinal epithelial cells (IECs) rather than that of hematopoietic cells led to LT + TNF-induced lethality. Inhibition of p38α mitogen-activated protein kinase (MAPK) signaling by LT in IECs promoted TNF-induced apoptosis and necroptosis of IECs, leading to intestinal damage and mouse death. Consistently, p38α inhibition by LT enhanced TNF-mediated cell death in human colon epithelial HT-29 cells. As intestinal damage is one of the leading causes of lethality in anthrax patients, the IEC damage caused by LT + TNF would most likely be a mechanism underneath this clinical manifestation and could be a target for interventions.

## Introduction

*Bacillus anthracis*, the causative agent of the serious infectious disease anthrax, is a Gram-positive bacterium that most frequently produces spores in soil. Accidental acquisition of the spores by mammals may lead to spore activation, generating anthrax bacteria that may penetrate the bloodstream, thereby initiating a systemic infection (Goel, 2015; Moayeri et al., 2015; Mock and Fouet, 2001; Sweeney et al., 2011). Mortality rates of anthrax vary in different types. For inhalation anthrax, it may exceed 90% (Holty et al., 2006; Huang et al., 2015). Symptoms of anthrax are complex and could include hemorrhagic necrosis of the lymph nodes, necrotizing pneumonia, hemorrhagic meningitis, and gastrointestinal submucosal hemorrhagic lesions (Abramova et al., 1993; Grinberg et al., 2001; Sirisanthana and Brown 2002; Twenhafel et al., 2007).

Anthrax toxins secreted by *B*. *anthracis* are composed of three proteins: protective antigen (PA), lethal factor (LF), and edema factor (EF) (Fish et al., 1968; Mock and Fouet, 2001; Stanley and Smith, 1961). A combination of PA + LF is called lethal toxin (LT) while a combination of PA + EF is called edema toxin (ET) (Firoved et al., 2005; Liu et al., 2013; Moayeri et al., 2003). The toxicity of LF depends on its protease activity specific towards several critical molecules related to cell survival and death, such as the mitogen-activated protein kinase (MAPK) kinases (MKKs or MEKs) (Duesbery et al., 1998), resulting in an impairment of MAPK activation (Bardwell et al., 2004). The p38 MAPK (MAPK14) pathway is one of the well-documented targets of LF whose inactivation sensitizes macrophages to inflammatory stimulus-induced cell death (Kim et al., 2003; Park et al., 2002). LF is also well known to cleave and activate the nucleotide-binding domain leucine-rich repeat receptor (NLR) family pyrin domain containing 1B (NLRP1B) in some mouse strains, leading to pyroptosis and lethality of these mice (Chui et al., 2019; Mitchell et al., 2019; Sandstrom et al., 2019). But NLRP1 in humans and NLRP1B in certain mouse strains such as C57BL/6 (B6) are resistant to LF cleavage, and macrophages from these mice are insensitive to LT-induced death *in vitro* (Boyden and Dietrich 2006). Since *B*. *anthracis* infection still kills these hosts (Moayeri et al., 2010; Terra et al., 2010), a common role of NLRP1/NLRP1B inflammasome in LT-caused pathogenesis in humans and mice has been excluded (Taabazuing et al., 2020).

Pathogenicity of *B*. *anthracis* relies on its special toxins but host responses towards bacterial infection are also indispensable. A key early pathogenic event that allows *B*. *anthracis* to establish infections has been demonstrated to be the targeting of myeloid cells by LT (Liu et al., 2010). Macrophage death was implicated to be important for this event and p38 pathway inhibition by LT plays a promoting role in cell death (Ali et al., 2011; Kim et al., 2003; Park et al., 2002; Van Hauwermeiren et al., 2022). As the infection progresses, LT, the dominant toxin, accumulates to high levels following the propagation of the bacteria (Mabry et al., 2006; Weiner et al., 2014). At a high dose, LT itself is toxic to animals, causing damage mainly to the cardiovascular system and subsequent mouse death (Liu et al., 2010, 2013). It is important to note that bacteria in the bloodstream also induce robust production of cytokines such as tumor necrosis factor-α (TNF) (Loving et al., 2007; Pickering et al., 2004), and a role of TNF in anthrax-caused death can be evidenced by the observation that anti-TNF antibody administration delays the death of B6 mice infected with *B*. *anthracis* (Kalns et al., 2002). Therefore, a combined effect of TNF and LT shall occur in *B*. *anthracis*-infected animals and be responsible for at least a part of the late-stage pathological changes caused by *B*. *anthracis*. However, the lethal mechanisms of this TNF + LT-driven pathology are largely unknown.

TNF is a pleiotropic pro-inflammatory cytokine that drives cytokine production/survival or cell death and thus is involved in many processes, including embryonic development (Zhang et al., 2021a) and sepsis (Zhang and Han 2022). p38 activation is one of the downstream events of TNF stimulation that leads to cell death blockade and pro-survival transcription (Dondelinger et al., 2017; Jaco et al., 2017; Lalaoui et al., 2016; Menon et al., 2017). When the survival pathway is blocked and receptor-interacting serine/threonine-protein kinase 3 (RIP3) is absent, TNF receptor 1 signaling leads to RIP1-FADD-caspase-8-mediated apoptosis; while in the presence of RIP3, RIP1-RIP3-mixed lineage kinase domain-like pseudokinase (MLKL) signaling could be activated (Chen et al., 2013; Cho et al., 2009; He et al., 2009; Huang et al., 2017; Morgan and Kim 2022; Wu et al., 2014; Yang et al., 2018; Zhang et al., 2009, 2017, 2018) and necroptosis occurs. It was reported recently that LT sensitizes TNF-induced activation of NLRP3 inflammasome and caspase-8-dependent apoptosis in macrophages (Van Hauwermeiren et al., 2022). Additionally, *Casp8* deficiency attenuated *B*. *anthracis-*induced lethality in *Rip3* knockout mice (Van Hauwermeiren et al., 2022), supporting the idea that TNF-activated apoptosis contributes to the lethal effect of anthrax toxins (Kim et al., 2003; Park et al., 2002).

In an effort to further study the effect of TNF + LT treatment *in vivo*, we found unexpectedly that intestinal epithelial cells (IECs) were targets of LT-induced death in the presence of TNF and the resultant intestinal damage played a pivotal role in the lethality of mice. Both necroptosis and apoptosis pathways participated in the TNF + LT-triggered IEC deaths and mouse death. Genetic deletion of *p38α* in IECs mimicked LT treatment in sensitizing small intestines to TNF-induced damage and mouse death, supporting the role of p38α inactivation in the pathology of anthrax. An implication of our data is that impairment of p38α and perhaps also other MAPK pathways in IECs by any natural means would make animals vulnerable to inflammation-caused tissue injury and animal death.

## Results

### Co-treatment of sub-lethal dose of LT and TNF induces intestinal tissue damage and mouse death

**Figure 1.**
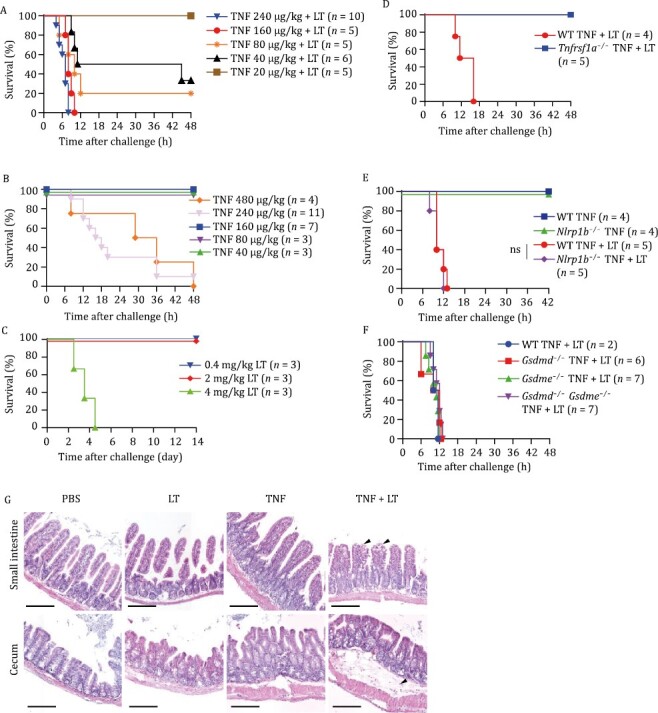
Co-treatment of sub-lethal dose of LT and TNF induces intestinal tissue damage and mouse deaths. (A–C) WT mice were intravenously (i.v.) injected with TNF at indicated doses and LT (0.4 mg/kg of LF and 0.4 mg/kg of PA, described as 0.4 mg/kg of LT hereinafter) (A), TNF alone (B), or LT alone (C), and monitored for survival rate. (D) WT mice and *Tnfrsf1a*^−/−^ mice were i.v. injected with TNF (160 μg/kg) and LT (0.4 mg/kg), and monitored for survival rate. TNF, LT, and TNF + LT for mouse treatment were used at this dosage from here on unless stated otherwise. (E and F) WT mice, *Nlrp1b*^−/−^ mice (E), *Gsdmd*^−/−^ mice, *Gsdme*^−/−^ mice, and *Gsdmd*^−/−^*Gsdme*^−/−^ mice (F) were i.v. injected with TNF-alone or in combination with LT, and monitored for survival rate. *P* values were calculated using a log-rank test (Mantel–Cox). ns, not significant. (G) WT mice were i.v. injected with TNF, LT, TNF + LT, or PBS. Mice were euthanized and tissues were collected 4 h after challenge. H&E staining of small intestines and ceca was shown. Arrowheads indicate damages. Scale bars, 200 μm. Data represent 4 mice per treatment pooled from two independent experiments.

Anthrax-caused lethality in B6 mice requires the LT secreted from *B*. *anthracis* bacteria, although the lethal mechanism is independent of NLRP1B cleavage (Boyden and Dietrich 2006; Chui et al., 2019; Mitchell et al., 2019; Moayeri et al., 2010; Sandstrom et al., 2019; Terra et al., 2010). Our previous study has shown that LT alone failed to induce B6 macrophage death while an addition of a low dose of TNF promoted macrophage death under LT stimulation (Kim et al., 2003). Both LT and TNF are present in the bloodstream and tissues of *B*. *anthracis*-infected mice (Loving et al., 2007; Mabry et al., 2006; Shemyakin et al., 2005; Weiner et al., 2014). Thus, we reasoned that a combinational treatment of LT + TNF in mice would be more clinically relevant than a single treatment of a high dose of LT or TNF in mimicking LT and TNF accumulation in the host. Similar to the cell-based results (Kim et al., 2003), co-stimulation of a sub-lethal dose of LT and TNF was sufficient to cause death in B6 mice (Fig. 1A–C). Examination of TNF concentration in the peripheral blood revealed that the amount of circulating TNF in the LT + TNF model was comparable with that in an lipopolysaccharide (LPS)-induced sepsis model (Fig. S1A) and was also commensurate with the records of murine or primate anthrax models (Shemyakin et al., 2005; Stearns-Kurosawa et al., 2006), supporting the appropriateness and clinical relevance of the LT + TNF model. *Tnfrsf1a*^*−/−*^ mice completely survived the challenge (Fig. 1D) while *Nlrp1b*^−/−^, *Gsdmd*^−/−^, *Gsdme*^−/−^, or *Gsdmd*^−/−^*Gsdme*^−/−^ mice were as sensitive as wild type (WT) to LT + TNF challenge (Fig. 1E and 1F). Thus, LT synergizes with TNF to drive mouse death in a TNF-signaling-dependent but NLRP1B/pyroptosis-independent manner.

High-dose LT-alone-induced mouse death was reported to be via targeting cardiomyocytes and vascular smooth muscle cells (Liu et al., 2013) while cecum was the initial damaged organ in TNF-alone-induced mouse death (Chen et al., 2015). Previous studies on LT + TNF treatment were mainly on macrophages (Ali et al., 2011; Kim et al., 2003) and *in vivo* tissue damages have not been examined. Unexpectedly, hematoxylin and eosin (H&E) staining analysis revealed that tissue damages in LT + TNF-treated mice were mainly in the small intestines and ceca (Fig. 1G), different from those observed in high-dose LT-treated mice or TNF-alone-induced mouse death. Colons, lungs, livers, kidneys, and spleens did not show significant damage although coagulation of blood could be observed in some of these tissues (Fig. S1B). Data obtained from intestinal organoid cultures also supported that LT increased the sensitivity of intestinal cells to TNF-induced death (Fig. S1C and S1D).

### Apoptosis and necroptosis pathways in IECs complement each other in mediating the death of B6 mice co-stimulated with TNF and LT

**Figure 2.**
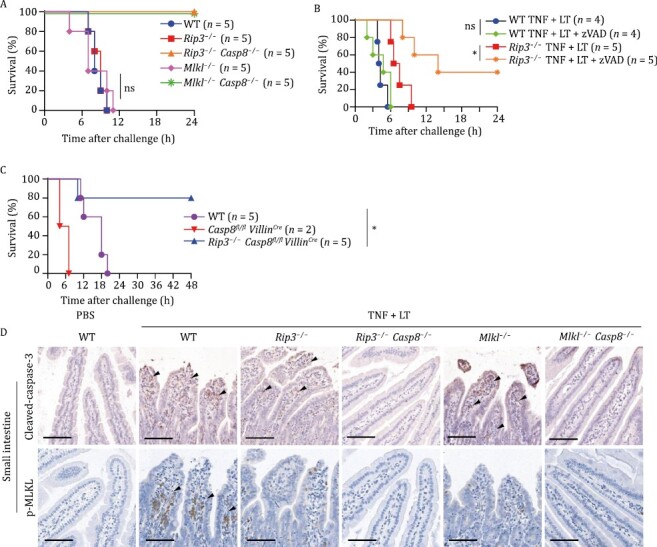
Apoptosis and necroptosis pathways in IECs complement each other in mediating death of B6 mice co-stimulated with TNF and LT. (A and D) Mice of indicated genotypes were i.v. injected with TNF plus LT. Survival rates were monitored (A) and IHC staining was performed for tissues collected 4 h after challenge (D). Scale bars, 100 μm. Images represent 3 mice per treatment pooled from two independent experiments. (B) WT and *Rip3*^−/−^ mice were i.v. injected with TNF plus LT and zVAD (10 mg/kg) or an equal volume of dimethyl sulfoxide (DMSO), and monitored for survival rate. (C) Mice of indicated genotypes were i.v. injected with TNF plus LT and survival rates were monitored. *P* values were calculated using a log-rank test (Mantel–Cox). *P < 0.05; ns, not significant.

It is known that TNF-induced B6 mouse death is primarily mediated by necroptosis at the minimal lethal dose and by both apoptosis and necroptosis when higher doses of TNF were applied (Duprez et al., 2011; Gunther et al., 2011; Newton et al., 2016; Vandenabeele et al., 2010) (Fig. S2A and S2B). However, when co-treated with a sub-lethal dose of TNF and LT, *Rip3*^−/−^ or *Mlkl*^−/−^ mice were as sensitive as WT to the challenge while *Rip3*^−/−^*Casp8*^−/−^ and *Mlkl*^−/−^*Casp8*^−/−^ mice were completely resistant (Fig. 2A), suggesting that TNF-induced apoptosis played a pivotal role in LT + TNF-induced mouse death. Nevertheless, the contribution of necroptosis still cannot be excluded. *Rip3*^−/−^ mice and *Mlkl*^−/−^ mice showed tissue damage in small intestines while no damage was found in small intestines of *Rip3*^−/−^*Casp8*^−/−^ or *Mlkl*^−/−^*Casp8*^−/−^ mice (Fig. S2C), suggesting a role of intestinal cell death in mediating LT + TNF-caused mouse death.

Since apoptosis and necroptosis pathways complement each other in mediating cell death (Han et al., 2011), we further evaluated whether this occurs in LT + TNF-induced mouse death. Given that *Casp8*^−/−^ mice die embryonically, we used a pan-caspase inhibitor z-VAD-FMK (zVAD) to illustrate the role of caspase-8 and found that neither zVAD alone nor *Rip3* knockout alone had any effect on LT + TNF-induced mouse death (Fig. 2B). Interestingly, zVAD attenuated the death of LT + TNF-treated *Rip3*^*−/−*^ mice (Fig. 2B), indicating that the switch between apoptosis and necroptosis occurred and that both apoptosis and necroptosis pathways participated in LT + TNF-induced mouse death by compensating each other.

Bone marrow transplantation revealed that loss of *Rip3* and *Casp8* in non-hematopoietic cells was sufficient to recapitulate the death-resistant phenotype of *Rip3*^−/−^*Casp8*^−/−^ mice while WT mice carrying *Rip3*^−/−^*Casp8*^−/−^ bone marrow died at similar kinetics to WT mice (Fig. S3A and S3B). Thus, RIP3- and caspase-8-mediated death pathways in non-hematopoietic cells but not hematopoietic cells played a predominant role in LT + TNF-induced animal death. Since damages were observed in intestinal villi (Figs. 1G and S2C), we then further narrowed the caspase-8-dependent cell death to IECs and found that mice with conditional deletion of *Casp8* in IECs (*Casp8*^*fl/fl*^*Villin*^*Cre*^) were more sensitive to LT + TNF-induced death than WT (Fig. 2C). Because *Casp8*^*fl/fl*^*Villin*^*Cre*^ mice had spontaneous ileitis and colitis (Gunther et al., 2011; Schwarzer et al., 2020), the increased mouse death might be due to the pre-presence of necroptotic damage of intestines in these mice. Indeed, additional deletion of *Rip3* eliminated the spontaneous ileitis and colitis (Gunther et al., 2011; Schwarzer et al., 2020) and rescued the death of *Casp8*^*fl/fl*^*Villin*^*Cre*^ mice under LT + TNF challenge (Fig. 2C). These data further supported the notion that complementation of apoptosis and necroptosis pathways occurred in the IECs of LT + TNF-treated mice.

Lactate dehydrogenase (LDH) release in serum is a readout of massive lytic cell death *in vivo*. LT + TNF-challenged WT mice exhibited a significant increase in serum LDH release (Fig. S3C), which was abolished by a combined loss of *Rip3* and *Casp8* or *Mlkl* and *Casp8* but not by a single knockout of *Rip3* or *Mlkl* (Fig. S3D), supporting a role of cell death in LT + TNF-induced animal death. Immunohistochemistry (IHC) staining for cleaved-caspase-3, a marker of apoptosis, and phosphorylated MLKL (p-MLKL), an indicator of necroptosis, in small intestines after LT + TNF challenge showed that apoptosis occurred in WT, *Rip3*^−/−^, or *Mlkl*^−/−^ mice but not in *Rip3*^−/−^*Casp8*^−/−^ or *Mlkl*^−/−^*Casp8*^−/−^ mice while necroptosis took place only in WT small intestines after challenge (Figs. 2D and S3E). Consistently, Western blot analysis showed caspase-8 cleavage in LT + TNF-challenged WT, *Rip3*^−/−^, or *Mlkl*^−/−^ IECs (Fig. S3F and S3G). In addition, no positive correlation between LT + TNF-induced mouse death and other major pro-inflammatory cytokines or eicosanoid mediators were observed (Figs. S4 and S5). Collectively, we concluded that both caspase-8-mediated apoptosis and RIP3-mediated necroptosis participate in LT + TNF-induced mouse death, and the intestine is the key organ target responsible for LT + TNF-induced tissue damage and subsequent animal death.

### LT via impairing the p38α pathway promotes TNF-induced cell death

**Figure 3.**
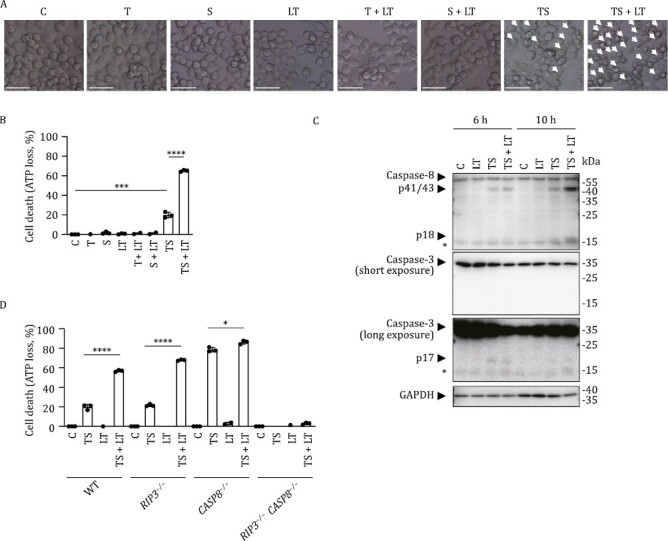
LT enhances TS-induced cell death in human HT-29 cells. (A–C) HT-29 cells were treated with TNF (T) (30 ng/mL), LCL-161 (S) (10 μmol/L), and/or LT (2 μg/mL of LF and 2 μg/mL of PA) for 12 h (A and B) or for indicated time points (C). Cell morphologies were shown (A), cell death was measured by ATP loss (B), and cell lysates were subjected to Western blot analysis (C). Scale bars, 50 μm. Images and Western blot analysis results represent technical triplicates pooled from two independent experiments. T, S, and LT were used at this dosage for HT-29 cells from here on unless stated otherwise. (D) HT-29 cells of indicated genotypes were treated with T, S, and/or LT for 12 h. Cell death was measured by ATP loss. *P* values were calculated using an unpaired Student's *t*-test. *P < 0.05; ***P < 0.001; ****P < 0.0001. Error bars represented the standard deviations of the means of technical triplicates pooled from two independent experiments (B and D).

LT alone kills BALB/c but not B6 macrophages by NLRP1B inflammasome activation (Fig. S6A–C) while LT + TNF efficiently induces death of macrophages from all mouse strains tested regardless of the forms of NLRP1B (Kim et al., 2003; Van Hauwermeiren et al., 2022). *In vivo* data confirmed that LT + TNF-induced IEC-mediated B6 mouse death was also NLRP1B/pyroptosis-independent (Fig. 1E and 1F). To study the molecular mechanisms underlying IEC death, we tested whether LT + TNF-induced IEC death can be reproduced in human colorectal adenocarcinoma cell line HT-29. It is known that inhibition of inhibitors of apoptosis (IAPs) by chemical inhibitors such as LCL-161 (S) promotes TNF-induced cell death in HT-29 cells (Fig. 3A and 3B) (Varfolomeev et al., 2007; Vince et al., 2007). In consistence with the *in vivo* observation (Fig. 1A and 1B), the addition of LT significantly enhanced TNF + S (TS)-caused cell death (Fig. 3A and 3B) and the cleavage of caspase-8 and caspase-3 (Fig. 3C), indicating that LT enhanced TS-induced apoptosis. In addition, LT-enhanced cell death was not blocked by deletion of either *CASP8* or *RIP3* but was completely abolished by a concomitant loss of *CASP8* and *RIP3* (Figs. 3D and S6D), supporting the notion that complementation between apoptosis and necroptosis occurred, similar to what happened in LT + TNF-induced epithelial cell death *in vivo* (Fig. 2A–D).

**Figure 4.**
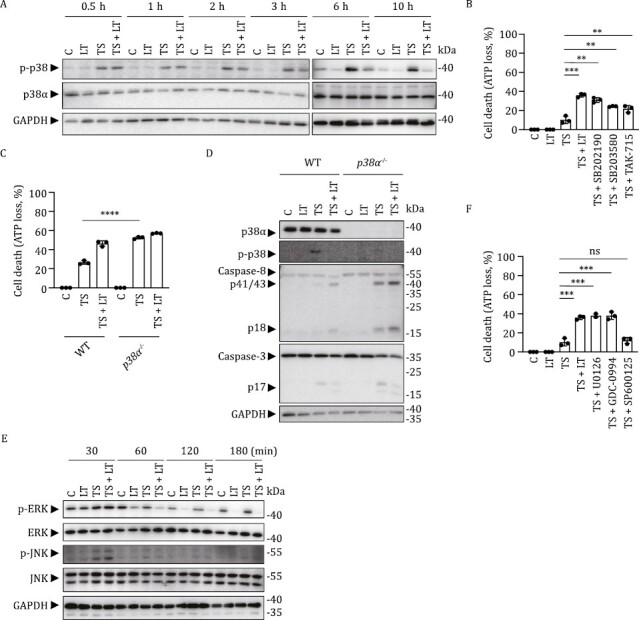
Inhibition of p38α enhances TS-induced cell death in HT-29 cells. (A) HT-29 cells were treated with TS and/or LT for indicated time periods and then cell lysates were subjected to Western blot analysis. (B) HT-29 cells were treated with TS with or without LT or p38α inhibitors (2 μmol/L of SB202190, 2 μmol/L of SB203580, or 2 μmol/L of TAK-715) for 12 h. Cell death was assessed by ATP loss. (C and D) WT and *p38α*^−/−^ HT-29 cells were treated with TS and/or LT for 12 h. Cell death was measured by ATP loss (C) and cell lysates were subjected to Western blot analysis (D). (E) HT-29 cells were treated with TS and/or LT for indicated time periods and cell lysates were subjected to Western blot analysis. (F) HT-29 cells were treated with TS with or without LT, ERK inhibitors (2 μmol/L of U0126 or 2 μmol/L of GDC-0994), or JNK inhibitor (10 μmol/L of SP600125) for 12 h. Cell death was measured by ATP loss. *P* values were calculated using an unpaired Student's *t*-test. **P < 0.01; ***P < 0.001; ****P < 0.0001. Error bars represented the standard deviations of the means of technical triplicates pooled from two independent experiments (B, C, and F). Western blot analysis results represent technical triplicates pooled from two independent experiments (A, D, and E).

The cleavage of MKKs by LF inhibits MAPK pathways, including p38, extracellular signal-regulated kinase (ERK), and c-Jun N-terminal kinase (JNK) (Ali et al., 2011; Bardwell et al., 2004; Fang et al., 2005; Raymond et al., 2010). Indeed, LT significantly inhibited p38α phosphorylation in TS-treated HT-29 cells (Fig. 4A). p38α inhibitors, SB202190, SB203580, and TAK-715, were all able to enhance TS-stimulated HT-29 cell death, phenocopying the TS + LT treatment (Fig. 4B). *p38α* knockout in HT-29 cells also increased TS-mediated cell death to an extent similar to that in TS + LT-treated WT cells (Fig. 4C). Furthermore, Western blot analysis revealed more cleaved-caspase-8 and cleaved-caspase-3 in *p38α*^−/−^ cells than in WT cells, indicating that TS-induced apoptosis was enhanced in the absence of p38α signaling (Fig. 4D). Moreover, *p38α* knockout eliminated most of the enhancing effect of LT on TS-induced cell death, caspase-8 cleavage, and caspase-3 cleavage (Fig. 4C and 4D), supporting that p38α signaling was a major downstream target of LT. LT also significantly inhibited ERK phosphorylation but not JNK phosphorylation in TS-stimulated HT-29 cells (Fig. 4E). ERK but not JNK inhibitors also increased death of TS-treated HT-29 cells, implying that ERK inhibition also contributed to LT-enhanced HT-29 cell death (Fig. 4F).

### p38α inhibition in IECs promotes TNF-induced B6 mouse death

**Figure 5.**
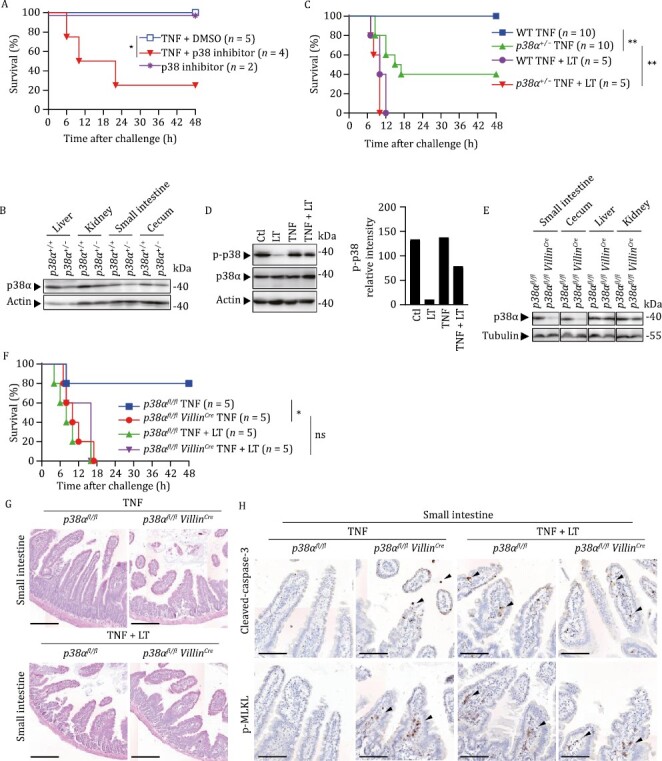
p38α inhibition in IECs promotes TNF-induced death in B6 mice. (A) WT mice were i.v. injected with TNF plus p38α inhibitor TAK-715 (10 mg/kg) or an equal volume of DMSO. Survival rates were monitored. (B and E) Tissue samples from mice of indicated genotypes were subjected to Western blot analysis. The results represent technical triplicates pooled from two independent experiments. (C) Mice of indicated genotypes were i.v. injected with TNF, with or without LT. Survival rates were monitored. (D) WT mice were i.v. injected with TNF, LT, or TNF + LT. Small intestinal epithelia were collected for Western blot analysis 4 h after challenge. The signal intensity of bands of interest was analyzed by ImageJ and normalized to that of Actin. Data represent technical triplicates pooled from two independent experiments. (F–H) Mice of indicated genotypes were i.v. injected with TNF, with or without LT. Survival rates were monitored (F). Small intestines were collected after euthanasia and subjected to H&E staining (G) and IHC staining (H) analysis. Scale bar, 200 μm in (G) and 100 μm in (H). Images represent technical triplicates pooled from two independent experiments. *P* values were calculated using a log-rank test (Mantel–Cox). *P < 0.05; **P < 0.01; ns, not significant.

In consistence with our *in vitro* study (Fig. 4B), injection of the p38α inhibitor TAK-715 caused death of B6 mice challenged with a sub-lethal dose of TNF (Fig. 5A). Since *p38α*^−/−^ mice are embryonically lethal, we tested the sensitivity of heterozygous *p38α*^+/−^ mice, whose p38α expression was lower than that in WT mice (Fig. 5B), to TNF-induced death. Loss of one allele of *p38α* significantly increased sensitivity of B6 mice to a sub-lethal dose of TNF, which was further enhanced by LT to a level similar to that of LT + TNF-treated WT mice (Fig. 5C). No synergistic effect on LT + TNF-caused mouse death by the reduction of *p38α* expression was observed, indicating that inhibition of p38α by LT sensitizes mice to TNF-induced death. In support of this notion, inhibition of p38α phosphorylation by LT was observed in isolated mouse IECs (Fig. 5D).

To examine whether p38α inhibition in the intestine by LT is the primary mechanism leading to the enhancement of TNF-induced mouse death, we generated *p38α*^*fl/fl*^*Villin*^*Cre*^ mice in which p38α expression was absent in small intestinal and cecal epithelial cells (Fig. 5E). Strikingly, *p38α*^*fl/fl*^*Villin*^*Cre*^ mice were as sensitive to TNF as *p38α*^*fl/fl*^ mice to LT + TNF and LT injection did not have a synergistic effect on the death of TNF-treated *p38α*^*fl/fl*^*Villin*^*Cre*^ mice (Fig. 5F), indicating that genetic depletion of p38α in IECs phenocopied the enhancement effect of LT on TNF-induced death. H&E staining showed that in sharp contrast to TNF-treated *p38α*^*fl/fl*^ mice, similar damages were observed in small intestines of TNF-treated *p38α*^*fl/fl*^*Villin*^*Cre*^ mice, LT + TNF-treated *p38α*^*fl/fl*^ mice, and LT + TNF-treated *p38α*^*fl/fl*^*Villin*^*Cre*^ mice (Fig. 5G), supporting the notion that the inhibition of p38α phosphorylation by LT in the IECs is the principal cause of the enhancement of TNF-induced lethality by LT. Furthermore, both caspase-3 cleavage and MLKL phosphorylation were detected in small intestines of TNF-treated *p38α*^*fl/fl*^*Villin*^*Cre*^ mice, LT + TNF-treated *p38α*^*fl/fl*^ mice, and LT + TNF-treated *p38α*^*fl/fl*^*Villin*^*Cre*^ mice (Fig. 5H), indicating that p38α inhibition by LT in IECs led to both apoptosis and necroptosis in small intestines. Collectively, our data revealed that the enhancement of TNF-induced mouse death by LT resulted primarily from an inhibition of TNF-induced p38α activation by LT in IECs, leading to an enhancement of TNF-induced apoptosis and necroptosis in IECs.

## Discussion

Pulmonary anthrax and gastrointestinal anthrax cause higher lethality than cutaneous anthrax in both animals and humans (Moayeri et al., 2015; Mock and Fouet 2001; Sweeney et al., 2011). Pathologically, gastrointestinal malfunctions were found not only in gastrointestinal anthrax patients but also in pulmonary and sometimes cutaneous cases. Intestinal hemorrhage is one of the leading causes of lethality (Abramova et al., 1993; Grinberg et al., 2001; Mock and Fouet 2001). In this study, we found that a synergistic effect of TNF and LT led to mouse death, featuring robust intestinal cell death and systemic LDH release (Figs. 1 and 2), a phenotype in consistence with clinical observations that gastrointestinal damage is one of the leading causes of the patient death.

LT is a key factor that determines the virulence of *B*. *anthracis*. LT targeting of myeloid cells *in vivo* is known to be essential for systemic dissemination of *B*. *anthracis* (Liu et al., 2010). However, host responses to *B. anthracis* such as cytokine secretion are not negligible and could participate in triggering host death. Indeed, a synergistic effect of LT and TNF has been reported in macrophages (Kim et al., 2003; Van Hauwermeiren et al., 2022). During mouse infection of *B*. *anthracis* or other bacteria, the amount of the pro-inflammatory cytokine TNF in the bloodstream increases to an average level of 200–1000 pg/mL (Shemyakin et al., 2005; Silva et al., 1990; Takashima et al., 1997; Yoshida et al., 2000), which is comparable with the serum TNF concentration in the TNF + LT model described in this work (Fig. S1A). Given the fact that TNF levels in the bloodstream of primates during infections can reach 10–40 ng/mL (Stearns-Kurosawa et al., 2006), the experimental setting of the current study using LT + TNF treatment has reasonably modeled an inflammatory response in *B. anthracis*-infected animals.

Further mechanistic study using various genetic tools showed that the key tissue target of LT + TNF was the small intestine and that the lethality was critically dependent on RIP3-MLKL-mediated necroptosis and caspase-8-mediated apoptosis in the intestine. Blocking either death pathway failed to prevent LT + TNF-mediated mouse death. Only concomitant genetic deletions of both pathways in the intestine completely prevented LT + TNF-mediated cell death, tissue damage, and animal death (Figs. 2 and 3). p38α inhibitors, *p38α* heterozygosity, or *p38α* conditional deficiency in IECs enhanced the sensitivity of mice to TNF, verifying that the intestine is the key organ responsible for LT + TNF-induced lethality and that p38α signaling in the intestine is the downstream target of LT, inhibiting RIP3- and caspase-8-mediated cell death in IECs (Fig. 5). *In vitro* data collected in human HT-29 cells (Figs. 3 and 4) and B6 macrophages (Kim et al., 2003) supported these conclusions obtained in B6 mice. We noticed that not only blocking p38α but also inhibiting ERK enhanced TNF-induced death of these cultured cells (Kim et al., 2003) (Fig. 4), but elimination of p38α signaling alone was sufficient to fully mimic the effect of LT on IECs in mice (Fig. 5F).

MAPK-activated protein kinase 2 (MK2) is a downstream effector kinase of p38. MK2 is reported to phosphorylate mouse RIP1 at S321 in response to TNF, resulting in RIP1 kinase inhibition, a failure to form RIP1-containing cytoplasmic cytotoxic complexes, and a blockage of RIP1-kinase-dependent necroptosis and apoptosis (Dondelinger et al., 2017; Jaco et al., 2017; Menon et al., 2017). Considering that an inhibition of p38 signaling promotes TNF-induced cell death and mouse death (Figs. 4 and 5), it is possible that LT inhibits p38 signaling, impairing MK2 activity and therefore enhancing TNF-induced RIP1-dependent necroptosis and apoptosis.

The cardiovascular system was reported to be the key tissue target responsible for the lethality caused by LT treatment alone (Liu et al., 2013). However, mice with IEC-specific deletion of the LT receptor capillary morphogenesis protein-2 (CMG2) or with IEC-specific CMG2-expression have not been tested for LT-induced lethality. When *B*. *anthracis* infection was considered, inflammatory cytokines produced by host cells, especially by the activated macrophages, contribute dramatically to the progression of lethal anthrax (Loving et al., 2007; Pickering et al., 2004). Therefore, a combinational challenge using LT + TNF and the conclusions generated using this model could be more relevant to bacterial infection than the treatment of LT or TNF alone. Indeed, our finding that the IECs are the key cell target of LT + TNF is reminiscent of the clinical evidence that intestinal damage is one of the leading causes of lethality in anthrax patients (Abramova et al., 1993; Grinberg et al., 2001; Mock and Fouet 2001).

LT-mediated killing of myeloid cells is essential for *B*. *anthracis* to paralyze host immune defenses and establish systemic dissemination (Liu et al., 2010). LT intoxication has recently been reported to sensitize macrophages to TNF-dependent NLRP3 inflammasome activation and apoptosis (Van Hauwermeiren et al., 2022). However, by bone marrow transplantation and the application of IEC conditional knockout mice, we showed here that the cell death of non-hematopoietic cells rather than hematopoietic cells is responsible for the lethality of LT + TNF-challenged mice, ruling out a role of LT-enhanced macrophage death in the lethality (Figs. 2C and S3A). Furthermore, we also provided genetic evidence elucidating that pyroptosis is not required for LT + TNF-induced animal death (Fig. 1F). But some mechanisms that were uncovered previously in macrophages also work in non-hematopoietic cells. For example, p38α inhibition by LT was reported to promote TNF-induced apoptosis in macrophages *in vitro* (Kim et al., 2003; Park et al., 2002; Van Hauwermeiren et al., 2022).

Global production of pro-inflammatory cytokines and lipid mediators is believed to be the major trigger of infection/inflammation-caused host death (Chousterman et al., 2017; Dennis and Norris 2015). However, we found that there is no correlation between host death and the levels of certain typical pro-inflammatory cytokines, such as interleukin (IL)-1β and IL-6, or lipid mediators in LT + TNF-challenged mice (Figs. S4 and S5). In contrast, our results showed a direct and strong correlation between host death and the levels of IEC death and tissue damage in LT + TNF-challenged mice (Figs. 1 and 2). These data demonstrated that TNF-induced cell death, both necroptosis and apoptosis, determines the lethality of challenged animals and supported our previous work that cell death, regardless of its type, plays a decisive role in animal death caused by NLRC4 inflammasome hyperactivation (Zhang et al., 2021b).

In short, by using a simplified but clinically relevant LT + TNF model, we found unexpectedly that IECs are key targeting cells responsible for LT-induced mouse death in the presence of TNF. Inhibiting p38α in the intestine by LT unleashes robust IEC death and intestinal damage caused by both RIP3-MLKL-mediated necroptosis and caspase-8-mediated apoptosis. Blocking either death pathway failed to prevent LT + TNF-mediated mouse death. Only deficiency of both death pathways in IECs completely prevented LT + TNF-caused cell death, tissue damage, and mouse death. Thus, preventing TNF-induced apoptosis and necroptosis in combination with controlling bacterial propagation might be an effective prevention of anthrax-caused death.

## Materials and methods

### Mice

*Nlrp1b* ^–/–^ mice, *Mlkl*^–/–^ mice, and *p38α*^+/–^ mice were generated using the CRISPR/Cas9 technology by Xiamen University Laboratory Animal Center as described previously (Zhong et al., 2015). gRNA targeting sequences were 5ʹ-AGGTTGTACTGCCATAGATGAGG-3ʹ and 5ʹ-TGACCCACCATAATACAAGCAGG-3ʹ for *Nlrp1b*, 5ʹ-GCACACGGTTTCCTAGACGC-3ʹ for *Mlkl*, and 5ʹ-AGGTCCGCCCCCATGAGAT-3ʹ for *p38α*. *Tnfrsf1a*^*−*/*−*^ mice, *Rip3*^*−*/−^ mice, *Casp8*^+/−^ mice, *Gsdmd*^−/−^ mice, and *Gsdme*^−/−^ mice were obtained as described before (He et al., 2015; Zhang et al., 2021a, 2021b). *Casp8*^*fl/fl*^ mouse strain was a kind gift from Dr. Stephen M. Hedrick (Beisner et al., 2005). *p38α*^*fl/fl*^ mice and *Villin*^*Cre*^ mice were from the Jackson Laboratory (JAX stock #031129 and #021504) (Otsuka et al., 2010). All knockout/knockin alleles have been crossed onto the B6 background, and mice with H19 and DMR mutations were excluded by using polymerase chain reaction (PCR) as reported previously (Zhong et al., 2015). The controls were sibling littermates. B6 male mice 8–12 weeks old were used in the study unless otherwise stated. All mice used in this work were housed under specific pathogen-free conditions with a 12-h light/dark cycle and had access to food and water *ad libitum* at Xiamen University Laboratory Animal Center. Before tissues were isolated, all mice were euthanized via CO_2_ exposure for at least 5 min until no breathing was observed. Death was ensured by performing a toe pinch. Cervical dislocation was performed as a secondary method of euthanasia.

### Generation of HT-29 knockout cell lines using the CRISPR/Cas9 technique

The gRNA targeting sequence was 5ʹ-CAGATCTGCCCCCATGAGAT-3ʹ for *p38α*, 5ʹ-TTAGTCACCAGAGCCGGCTT-3ʹ for *RIP3*, and 5ʹ-CCTGGACTACATTCCGCAA-3ʹ for *CASP8*. Plasmids harboring the gRNA sequence and Cas9 gene were transfected into HEK293T cells together with lentivirus-packaging plasmids, media were changed 12 h later and the supernatants were collected after 48 h. The viruses were then used to infect HT-29 cells. Disruption of the target gene was determined by immunoblots and further confirmed by sequencing.

### Animal challenge

Mice were randomly assigned to the control or experimental groups. Mouse TNF (CF09, Novoprotein, China) and/or LT [LF (172C, List Biological Labs, USA) and PA (171E, List Biological Labs)] diluted in 200 μL of phosphate-buffered saline (PBS) were intravenously injected into the mouse tail-vein with or without inhibitors of caspase (Z-VAD-FMK, 627610, Calbiochem, Germany) or p38α (TAK-715, HY-10456, MedChemExpress, USA) diluted at indicated doses. Mouse survival was monitored and recorded at indicated time points.

### Bone marrow transplantation

Six-week-old recipient mice were irradiated using an irradiator (RS 2000 Pro, Rad Source Technologies, USA) at a dose of 8 Gray. Bone marrow cells were isolated from femurs, tibias, and humeri of the donor mice, and erythrocytes were lysed by ACK lysis buffer (C3702, Beyotime, China). Bone marrow cells (5 × 10^6^ cells) were intravenously injected into each recipient mouse 4 h after irradiation. The chimerism of the recipient mice was examined 2 months later by PCR analysis of the genomic DNA extracted from the ear pinna tissue and the peripheral blood leukocytes for genotyping non-hematopoietic cells and hematopoietic cells, respectively.

### Cell culture and treatment

Peritoneal macrophages were prepared as described previously (Zhang *et al.* 2021b) and were cultured in RPMI 1640 medium supplemented with 10% FBS (SH30071.03, HyClone, USA), 100 units/mL of penicillin, and 100 μg/mL of streptomycin. 2 μg/mL of LT [2 μg/mL of LF (172C, List Biological Labs) and 2 μg/mL of PA (171E, List Biological Labs)] was used to treat peritoneal macrophages for 3 h. HT-29 cells of indicated genotypes were cultured in DMEM supplemented with 10% FBS (10099-141, Gibco, USA), 100 units/mL of penicillin, and 100 μg/mL of streptomycin. 2 μg/mL of LT, 30 ng/mL of human TNF (PHC3011, Gibco), and 10 μmol/L of LCL-161 were used to induce cell death in HT-29 cells. Inhibitors used in this study were 2 μmol/L of SB202190 (S1077, Selleck, USA), 2 μmol/L of SB203580 (559389, Calbiochem), 2 μmol/L of TAK-715 (HY-10456, MedChemExpress), 2 μmol/L of U0126 (S1102, Selleck), 2 μmol/L of GDC-0994 (S7554, Selleck), and 10 μmol/L of SP600125 (S5567, Sigma–Aldrich, USA).

### Detection of cell viability

The CellTiter-Glo Luminescent Cell Viability Assay Kit (G7571, Promega, USA) was used according to the manufacturer’s instructions.

### Immunoblot analysis

Immunoblot was performed as described previously (Zhang et al., 2021b). Antibodies used included: mouse caspase-1 (clone 4B4) [a kind gift from V. M. Dixit (Genentech, USA)], mouse GSDMD (ab209845, Abcam, UK), pro-caspase-8 (4790, Cell Signaling Technology, USA), mouse cleaved-caspase-8 (9429, Cell Signaling Technology), mouse RIP3 (homemade), mouse MLKL (homemade), human caspase-8 (ALX-804-242-C100, Enzo Life Sciences, USA), caspase-3 (9662, Cell Signaling Technology), human RIP3 (13526, Cell Signaling Technology), p38α (9228, Cell Signaling Technology), p-p38 (9211, Cell Signaling Technology), ERK (9107, Cell Signaling Technology), p-ERK (4377, Cell Signaling Technology), JNK (9252, Cell Signaling Technology), p-JNK (9251, Cell Signaling Technology), mouse p38α (homemade), Tubulin (M20005M, Abmart, China), Actin (homemade), and GAPDH (AC002, ABclonal, China).

### H&E staining

Animals were euthanized. Tissues were collected and fixed immediately in 4% paraformaldehyde for 24 h. The fixed tissues were embedded in a waterproof condition. Five-micrometer sections were obtained and stained with H&E. Slides were analyzed on the Leica Aperio Versa 200 (Leica Biosystems, Germany) (He et al., 2021; Li et al., 2022).

### IHC staining

Phosphorylated MLKL staining was conducted as described previously (He et al., 2021). For cleaved-caspase-3 staining, sections were treated using the Avidin/Biotin Blocking Kit (SP-2001, Vector Laboratories, USA) after being soaked in 3% hydrogen peroxide. VECTASTAIN Elite ABC-HRP Kit (PK-6100, Vector Laboratories) was used after antibody incubation. Antibodies used included: cleaved-caspase-3 (9661, Cell Signaling Technology), p-MLKL (ab196436, Abcam), Rabbit IgG, biotinylated (BA-1000, Vector Laboratories).

### Enzyme-linked immunosorbent assay

Cytokine levels [TNF, IL-1β, IL-6, monocyte chemotactic protein 1 (MCP-1), and interferon (IFN)-γ] in mouse sera were determined by using the MILLIPLEX MAP Mouse Cytokine/Chemokine Magnetic Bead Panel-Immunology Multiplex Assay (MCYTOMAG-70K, Millipore, USA) according to the manufacturer’s instructions.

### Eicosanoid analysis

Mouse blood was collected and 50 μL of the sera was immediately transferred to 500 μL of cold methanol for storage at −80°C. Eicosanoids and docosanoids were identified and quantified as described previously (Zhang et al., 2021b).

### Organoid culture

Organoid culture and challenge were performed as described previously (Wang et al., 2020). Organoids were observed at indicated time points. Cell death was measured by propidium iodide staining.

### Statistics

GraphPad Prism 5.0 software (GraphPad Software, USA) was used for data analysis. The log-rank test (Mantel–Cox) was used to compare the survival curves of mice. Error bars represented the standard deviations of the means of technical triplicates pooled from two independent experiments.

## Supplementary Material

pwad050_suppl_Supplementary_Figures_S1-S6

## Data Availability

The datasets generated during and/or analyzed during the current study are available from the corresponding author upon reasonable request.

## Abbreviations

B6: C57BL/6
*B*. *anthracis*: *Bacillus anthracis*
EF: edema factor
ERK: extracellular signal-regulated kinase
ET: edema toxin
H&E: hematoxylin and eosin
IAPs: inhibitors of apoptosis
IECs: intestinal epithelial cells
IHC: immunohistochemistry
JNK: c-Jun N-terminal kinase
LDH: lactate dehydrogenase
LF: lethal factor
LT: lethal toxin
MAPK: mitogen-activated protein kinase
MK2: mitogen-activated protein kinase-activated protein kinase 2
MKK: mitogen-activated protein kinase kinases
MLKL: mixed lineage kinase domain like pseudokinase
NLRP1B: NLR family pyrin domain containing 1B
p38: p38 mitogen-activated protein kinase
PA: protective antigen
p-MLKL: phosphorylated MLKL
RIP1: receptor-interacting serine/threonine-protein kinase 1
RIP3: receptor-interacting serine/threonine-protein kinase 3
TNF: tumor necrosis factor-α
TS: TNF + Smac-mimetic LCL-161
WT: wild type
zVAD: z-VAD-FMK
