# Supplementary material for: Anthrax lethal toxin and tumor necrosis factor-α synergize on intestinal epithelia to induce mouse death
Source: Protein Cell. 2023 Oct 19;15(2):135–48. doi: 10.1093/procel/pwad050 (PMC10833652; doi:10.1093/procel/pwad050)
Supplement: pwad050_suppl_Supplementary_Figures_S1-S6 [file pwad050_suppl_supplementary_figures_s1-s6.pdf]

SUPPLEMENTARY FIGURE LEGENDS

Supplementary Fig. 1. Co-treatment of sub-lethal dose of TNF+LT induced intestinal tissue damages.

(A) WT mice were i.v. injected with PBS, TNF, LT, or TNF+LT, or intraperitoneally (i.p.) injected with lipopolysaccharide (LPS) (50 mg/kg). Mice were euthanized and sera were collected for measurement of TNF 4 hours after challenge. LPS treatment was included as a positive control. Error bars represented the standard deviations of the means of technical triplicates pooled from two independent experiments. *P* values were calculated using an unpaired Student *t* test. ns, not significant.

(B) WT mice were i.v. injected with TNF, LT, TNF+LT, or PBS. Mice were euthanized and tissues were collected 4 hours after challenge. H&E staining of indicated tissues was shown. Scale bars, 200 μm or 100 μm as indicated. Images represent 4 mice per treatment pooled from two independent experiments.

(C and D) Small intestinal organoids from WT mice were treated with TNF (10 ng/mL) with or without LT (2 μg/mL of LF and 2 μg/mL of PA). Representative organoid images were taken at indicated time points after treatment (scale bar, 100 μm) (C) and percentages of PI-positive organoids were calculated (D). *P* values were calculated using an unpaired Student *t* test. ***, $P < 0.001$; ns, not significant. Error bars represented the standard deviations of the means of technical triplicates pooled from two independent experiments.

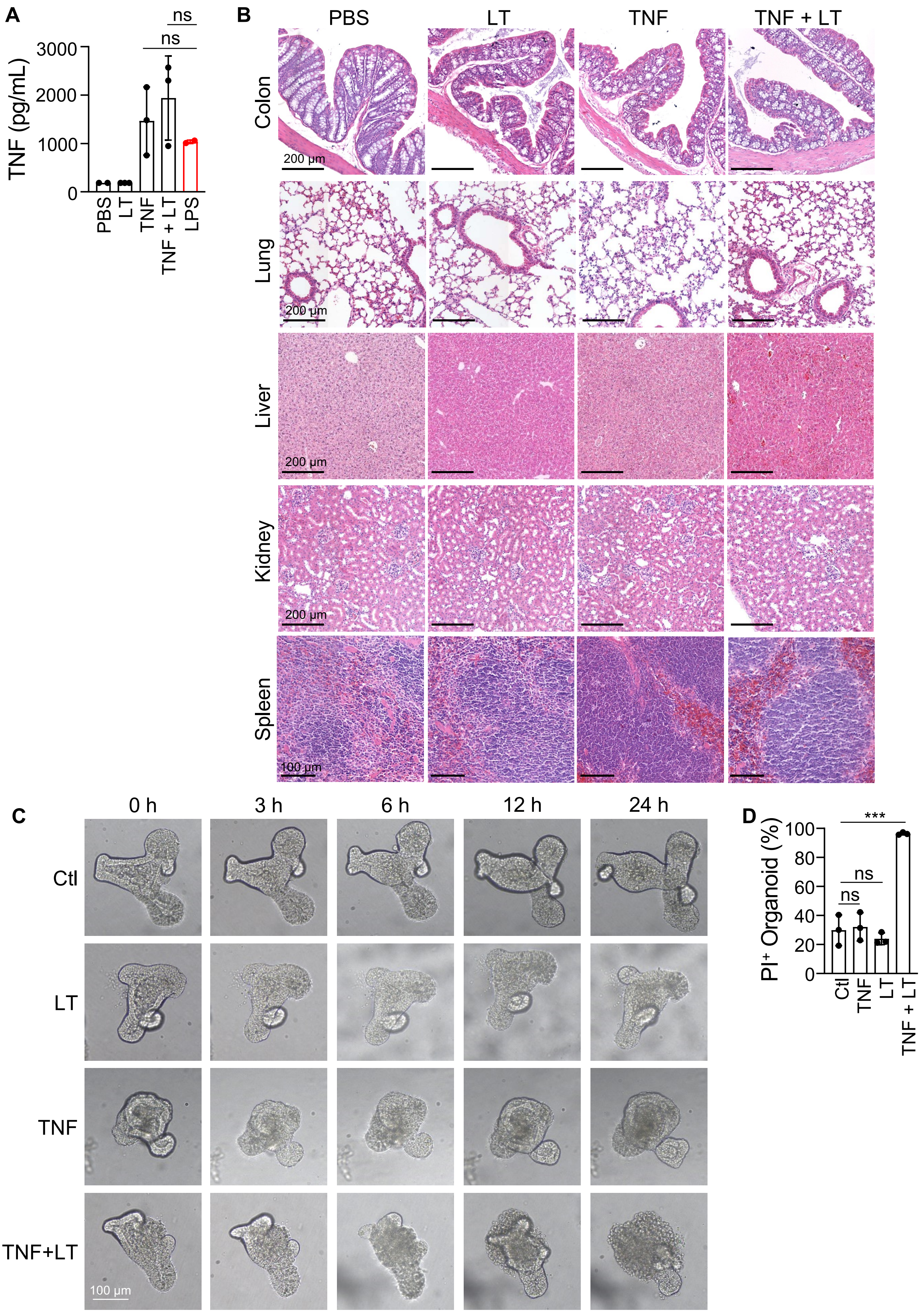

A
TNF (pg/mL)
3000
2000
1000
0
ns
ns
PBS
LT
TNF
TNF + LT
LPS
B
PBS
LT
TNF
TNF + LT
Colon
Lung
Liver
Kidney
Spleen
200 μm
100 μm
C
0 h
3 h
6 h
12 h
24 h
Ctl
LT
TNF
TNF+LT
100 μm
D
PI+ Organoid (%)
100
80
60
40
20
0
***
ns
ns
Ctl
TNF
LT
TNF + LT

Supplementary Fig. 2. Apoptosis and necroptosis pathways complement each other in mediating death of B6 mice co-stimulated with TNF and LT.

(A and B) Mice of indicated genotypes were i.v. injected with TNF (240 μg/kg in (A) and 1200 μg/kg in (B)) and monitored for survival rate.

(C) Mice of indicated genotypes were i.v. injected with TNF plus LT. Mice were euthanized and tissues were collected 4 hours after challenge. H&E staining was applied in small intestines. Scale bars, 200 μm. Images represent 3 mice per treatment pooled from two independent experiments.

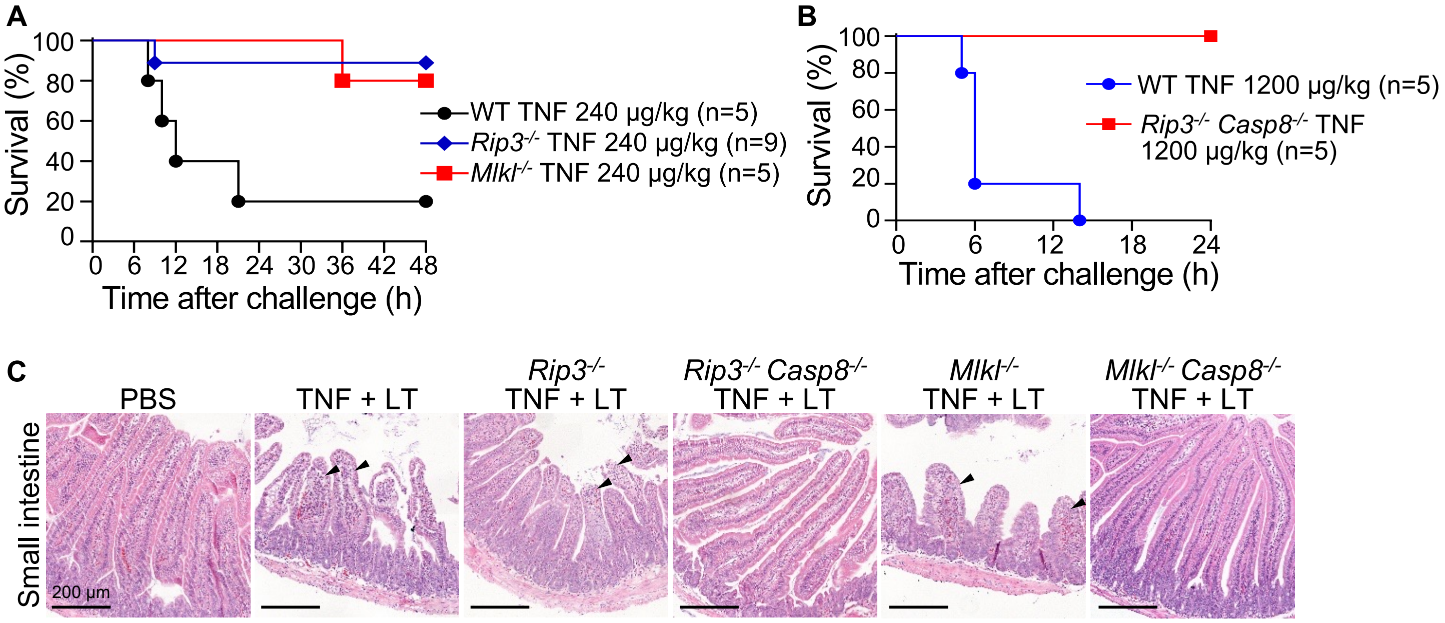
A
Survival (%)
100
80
60
40
20
0
0 6 12 18 24 30 36 42 48
Time after challenge (h)
WT TNF 240 µg/kg (n=5)
Rip3-/- TNF 240 µg/kg (n=9)
Mlkl-/- TNF 240 µg/kg (n=5)
B
Survival (%)
100
80
60
40
20
0
0 6 12 18 24
Time after challenge (h)
WT TNF 1200 µg/kg (n=5)
Rip3-/- Casp8-/- TNF 1200 µg/kg (n=5)
C
PBS
TNF + LT
Rip3-/- TNF + LT
Rip3-/- Casp8-/- TNF + LT
Mlkl-/- TNF + LT
Mlkl-/- Casp8-/- TNF + LT
Small intestine
200 µm

Supplementary Fig. 3. RIP3- and Caspase-8-dependent signaling in intestinal epithelial cells determines LT+TNF-induced animal death.

(A) WT or *Rip3*$^{-/-}$*Casp8*$^{-/-}$ recipient mice were irradiated and transplanted with bone marrow cells of indicated genotypes. TNF plus LT was subsequently i.v. injected into recipient mice and survival rates were monitored.

(B) Genotyping of chimeric mice after bone marrow transplantation. KO, *Rip3* or *Casp8* knockout. DKO, *Rip3* and *Casp8* double knockout. * stands for the mouse which was not irradiated successfully and thus was excluded from the TNF+LT-challenge.

(C and D) WT mice (C) or mice of indicated genotypes (D) were i.v. injected with TNF, LT, TNF+ LT, or PBS. Mice were euthanized and tissues were collected 4 hours after challenge and lactate dehydrogenase (LDH) release in mouse serum was measured. Sera from mice i.p. injected with 50 mg/kg of LPS for 4 hours were included as positive controls. Error bars represented the standard deviations of the means of technical triplicates pooled from two independent experiments. P values were calculated using an unpaired Student t test. *, $P < 0.05$; ***, $P < 0.001$; ns, not significant.

(E) WT mice were i.v. injected with TNF, LT, TNF+LT, or PBS. Mice were euthanized and tissues were collected 4 hours after challenge. Immunohistochemistry (IHC) staining of small intestines for cleaved-caspase-3 and phospho-MLKL was performed. Scale bars, 100 μm. Images represent 4 mice per treatment pooled from two independent experiments.

(F and G) WT mice (F) or mice of indicated genotypes (G) were i.v. injected with TNF, LT, TNF+ LT, or PBS. Small intestinal epithelia were isolated 4 hours post-challenge and subjected to western blot. Data represent 3 mice per treatment pooled from two independent experiments.

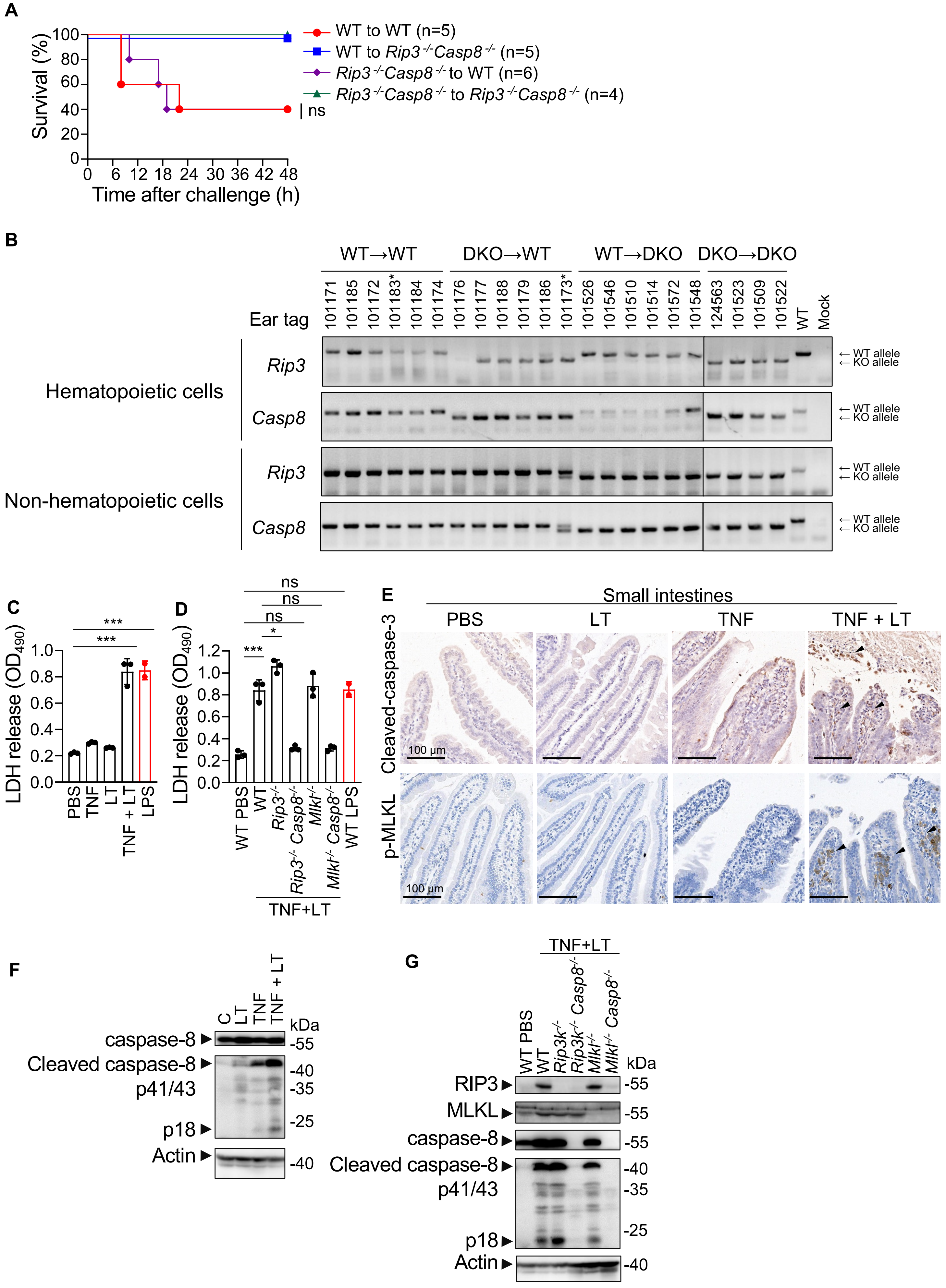
A
Survival (%)
Time after challenge (h)
WT to WT (n=5)
WT to Rip3-/-Casp8-/- (n=5)
Rip3-/-Casp8-/- to WT (n=6)
Rip3-/-Casp8-/- to Rip3-/-Casp8-/- (n=4)
ns
B
WT→WT
DKO→WT
WT→DKO
DKO→DKO
Ear tag
101171 101185 101172 101183* 101184 101174 101176 101177 101188 101179 101186 101173* 101526 101546 101510 101514 101572 101548 124563 101523 101509 101522 WT Mock
Hematopoietic cells
Non-hematopoietic cells
Rip3
Casp8
← WT allele
← KO allele
C
LDH release (OD490)
PBS TNF LT TNF + LT LPS
D
WT PBS WT Rip3-/- Rip3-/- Casp8-/- Mlkl-/- Mlkl-/- Casp8-/- WT LPS
TNF+LT
E
Small intestines
PBS LT TNF TNF + LT
Cleaved-caspase-3
p-MLKL
100 µm
F
C LT TNF TNF + LT
kDa
caspase-8
Cleaved caspase-8
p41/43
p18
Actin
G
TNF+LT
WT PBS WT Rip3k-/- Rip3k-/- Casp8-/- Mlkl-/- Mlkl-/- Casp8-/-
RIP3
MLKL
caspase-8
Cleaved caspase-8
p41/43
p18
Actin

Supplementary Fig. 4. Changes in cytokines or lipid mediators do not correlate with TNF+LT-induced mouse death.

WT mice were i.v. injected with TNF, LT, TNF+LT, or PBS. Mice were euthanized and sera were collected for measurement of cytokines and lipid mediators 4 hours after challenge. Sera from mice i.p. injected with 50 mg/kg of LPS for 4 hours were included as positive controls (A to D). Error bars represented the standard deviations of the means of technical triplicates pooled from two independent experiments.

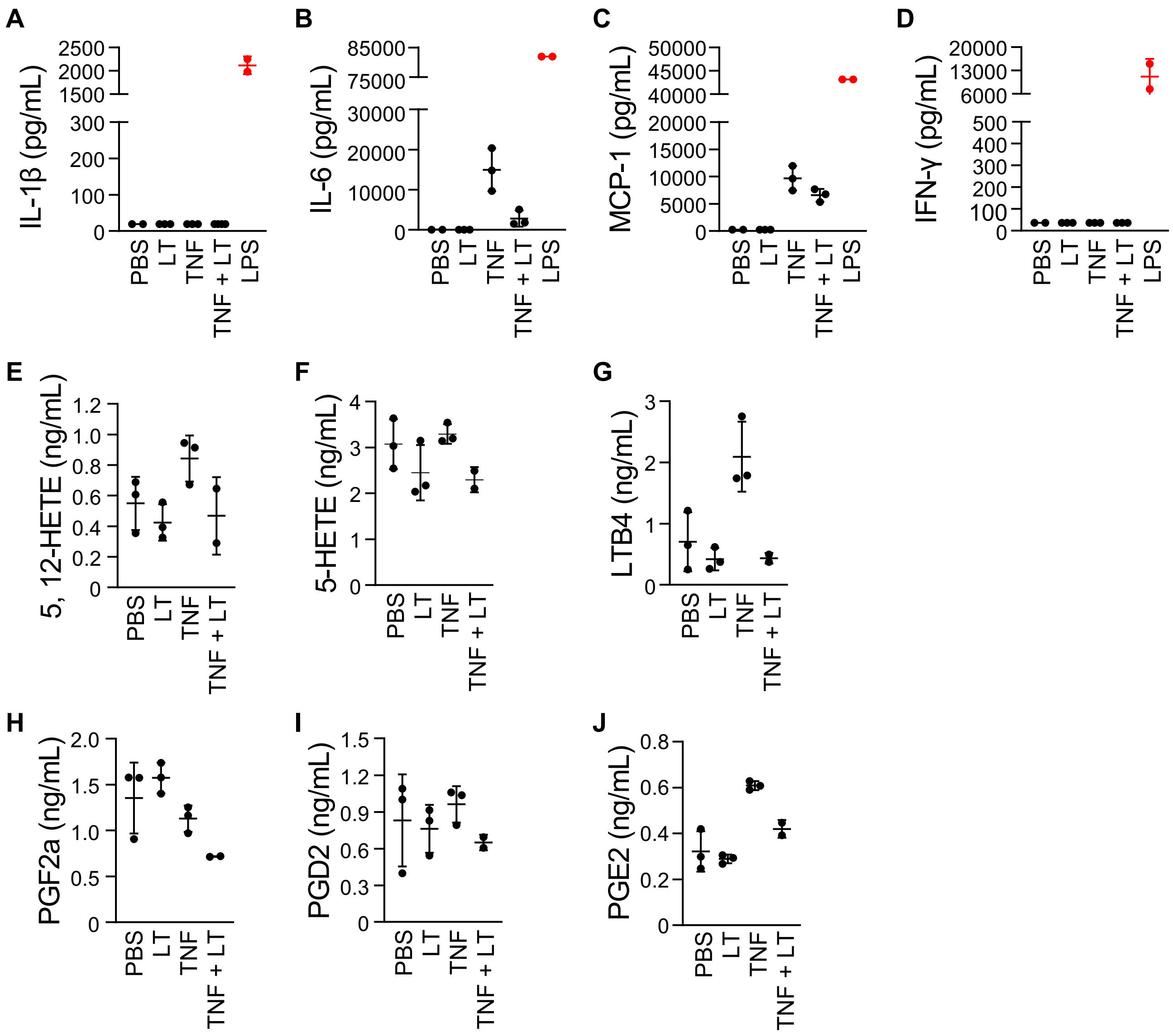
A
IL-1β (pg/mL)
2500
2000
1500
300
200
100
0
PBS
LT
TNF
TNF + LT
LPS
B
IL-6 (pg/mL)
85000
75000
30000
20000
10000
0
PBS
LT
TNF
TNF + LT
LPS
C
MCP-1 (pg/mL)
50000
45000
40000
20000
15000
10000
5000
0
PBS
LT
TNF
TNF + LT
LPS
D
IFN-γ (pg/mL)
20000
13000
6000
500
400
300
200
100
0
PBS
LT
TNF
TNF + LT
LPS
E
5, 12-HETE (ng/mL)
1.2
1.0
0.8
0.6
0.4
0.2
0
PBS
LT
TNF
TNF + LT
F
5-HETE (ng/mL)
4
3
2
1
0
PBS
LT
TNF
TNF + LT
G
LTB4 (ng/mL)
3
2
1
0
PBS
LT
TNF
TNF + LT
H
PGF2a (ng/mL)
2.0
1.5
1.0
0.5
0
PBS
LT
TNF
TNF + LT
I
PGD2 (ng/mL)
1.5
1.2
0.9
0.6
0.3
0
PBS
LT
TNF
TNF + LT
J
PGE2 (ng/mL)
0.8
0.6
0.4
0.2
0
PBS
LT
TNF
TNF + LT

Supplementary Fig. 5. Changes in cytokines or lipid mediators do not correlate with resistance of *Rip3*$^{-/-}$ *Casp8*$^{-/-}$ or *Mlkl*$^{-/-}$ *Casp8*$^{-/-}$ mice to TNF+LT-induced death.

Mice of indicated genotypes were i.v. injected with TNF plus LT. Mice were euthanized and sera were collected for measurement of cytokines and lipid mediators 4 hours after challenge. Sera from mice i.p. injected with 50 mg/kg of LPS for 4 hours were used as positive controls (A to D). Error bars represented the standard deviations of the means of technical triplicates pooled from two independent experiments.

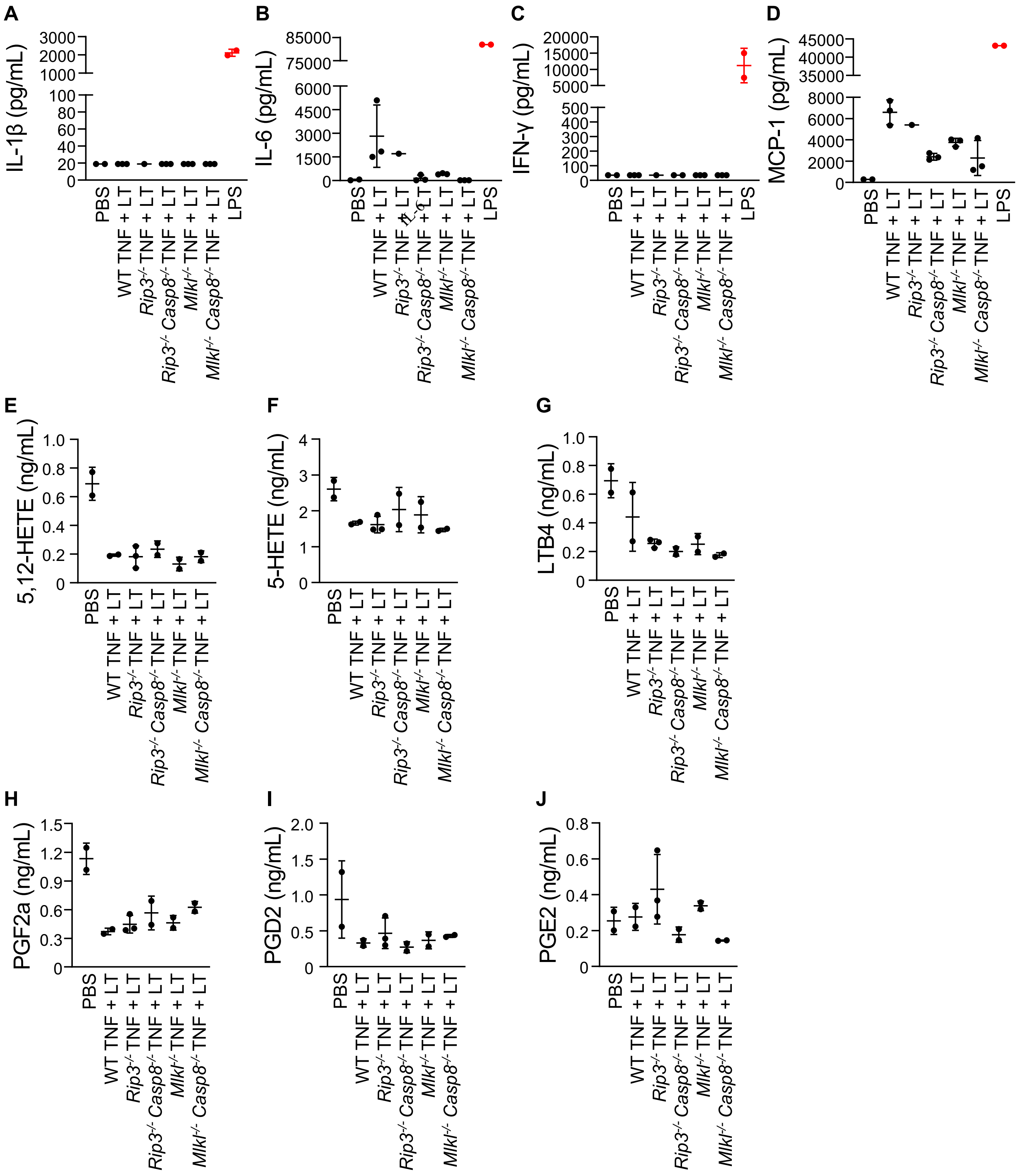

A
IL-1β (pg/mL)
B
IL-6 (pg/mL)
C
IFN-γ (pg/mL)
D
MCP-1 (pg/mL)
E
5,12-HETE (ng/mL)
F
5-HETE (ng/mL)
G
LTB4 (ng/mL)
H
PGF2a (ng/mL)
I
PGD2 (ng/mL)
J
PGE2 (ng/mL)
PBS
WT TNF + LT
Rip3-/- TNF + LT
Rip3-/- Casp8-/- TNF + LT
Mlkl-/- TNF + LT
Mlkl-/- Casp8-/- TNF + LT
LPS

Supplementary Fig. 6. LT induces pyroptosis in peritoneal macrophages of BALB/c but not B6 mice.

(A to C) Peritoneal macrophages from B6 or BALB/c mice were treated with LT (2 μg/mL of LF and 2 μg/mL of PA) for 3 hours. ATP loss (A) and LDH release (B) were measured. Error bars represented the standard deviations of the means of technical triplicates pooled from two independent experiments. Cell lysates were subjected to western blot (C).

(D) Cell lysates of HT-29 cells of indicated genotypes were subjected to western blot. Western blot results represent technical triplicates pooled from two independent experiments.

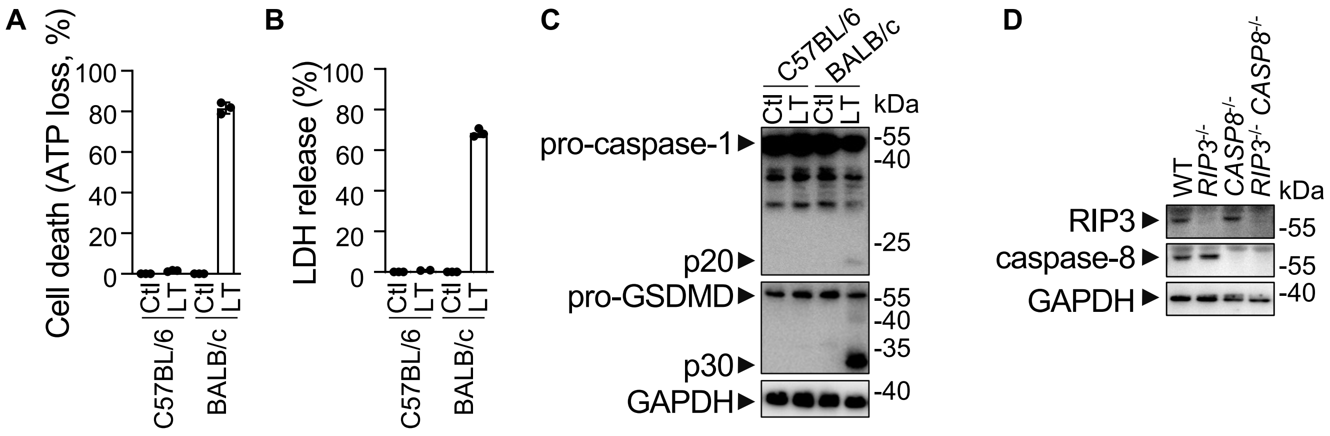
A
Cell death (ATP loss, %)
0
20
40
60
80
100
Ctl
LT
Ctl
LT
C57BL/6
BALB/c
B
LDH release (%)
0
20
40
60
80
100
Ctl
LT
Ctl
LT
C57BL/6
BALB/c
C
C57BL/6
BALB/c
Ctl
LT
Ctl
LT
kDa
pro-caspase-1
p20
pro-GSDMD
p30
GAPDH
-55
-40
-25
-55
-40
-35
-40
D
WT
RIP3-/-
CASP8-/-
RIP3-/- CASP8-/-
kDa
RIP3
caspase-8
GAPDH
-55
-55
-40
